# Functional Connectivity Changes Associated With Depression in Dementia With Lewy Bodies

**DOI:** 10.1002/gps.70058

**Published:** 2025-02-26

**Authors:** Manon Querry, Anne Botzung, Marion Sourty, Elena Chabran, Léa Sanna, Paulo Loureiro de Sousa, Benjamin Cretin, Catherine Demuynck, Candice Muller, Alix Ravier, Benoît Schorr, Nathalie Philippi, Frédéric Blanc

**Affiliations:** ^1^ University of Strasbourg and CNRS ICube Laboratory UMR 7357 and FMTS (Fédération de Médecine Translationnelle de Strasbourg) IMIS Team Strasbourg France; ^2^ Geriatrics Division University Hospitals of Strasbourg CM2R (Research and Resources Memory Centre) Geriatric Day Hospital Strasbourg France

**Keywords:** antidepressant treatment, dementia with Lewy bodies, depression, fMRI, salience network

## Abstract

**Objectives:**

Depressive symptoms are frequent in the early stages of dementia with Lewy bodies (DLB), and more than half of DLB patients would have a history of depression. Our study sought to investigate the functional connectivity (FC) changes associated with depressive symptoms in prodromal to mild DLB patients compared with controls.

**Methods:**

MRI data were collected from 66 DLB patients and 18 controls. Depression was evaluated with the Mini International Neuropsychiatric Interview. Resting‐state FC (rsFC) was investigated with the CONN toolbox using a seed‐based approach and both regression and comparison analyses.

**Results:**

Correlations were found between the depression scores and the rsFC between fronto‐temporal and primary visual areas in DLB patients (*p* < 0.05, FDR corrected). Depressed DLB patients also showed decreased rsFC within the salience network (SN), increased rsFC between the default mode network (DMN) and the language network (LN) and decreased rsFC between the cerebellar network (CN) and the fronto‐parietal network (FPN) compared to non‐depressed DLB patients (*p* < 0.05, uncorrected). Comparison analyses between antidepressant‐treated and non‐treated DLB patients highlighted FC changes in treated patients involving the SN, the DMN, the FPN and the dorsal attentional network (*p* < 0.05, uncorrected).

**Conclusions:**

Our findings revealed that depressive symptoms would especially be associated with rsFC changes between fronto‐temporal and primary visual areas in DLB patients. Such alterations could contribute to difficulties in regulating emotions, processing biases towards negative stimuli, and self‐focused ruminations.

**Trial Registration:**

This study is part of the cohort study AlphaLewyMA (https://clinicaltrials.gov/ct2/show/NCT01876459)

**Summary**

- In prodromal to mild DLB patients, depressive symptoms are associated with specific alterations in functional connectivity, particularly between fronto‐temporal regions and visual primary areas.
- The salience network may be an important functional network in understanding the close link between DLB and depression.
- The functional connectivity changes observed in DLB patients could specifically contribute to cognitive bias towards negative information, ruminations and emotional dysregulation.
- Antidepressant treatment might help to modulate the brain functional connectivity in DLB patients with depressive symptoms.

## 1 Introduction

Dementia with Lewy bodies (DLB) is the second most common form of neurodegenerative disease after Alzheimer's disease (AD). DLB is one of the synucleinopathies, diseases that are characterised by a diffuse aggregation of abnormal *α*‐synuclein. In addition to cognitive impairment, DLB is defined by the presence of at least two of the following core criteria: cognitive fluctuations, recurrent visual hallucinations, spontaneous parkinsonian features, and rapid eye movement sleep behaviour disorder (RBD) [1, 2]. Additionally, depression has been reported to be a supportive clinical feature of DLB [1, 2]. Depressive symptoms would affect between 34% [3] and 55% [4] of DLB patients, and 57% of DLB patients would have a history of depression [5].

Lately, there has been growing interest in brain functional connectivity (FC) and its relationship to the pathophysiology of symptoms in DLB [6]. Although showing heterogeneous results, the literature suggests that the disease is generally associated with disruptions in resting‐state functional connectivity (rsFC), especially within the salience network (SN) [7, 8], the fronto‐parietal network (FPN) [7, 8, 9, 10] and the default mode network (DMN) [8, 9, 11, 12]. However, to our knowledge, no study has yet looked specifically at the FC changes associated with depressive symptoms in DLB. A meta‐analysis nevertheless suggested the potential involvement of the FPN, the DMN, the ventral attentional network (VAN) and the limbic network in the affective symptomatology observed in *α*‐synucleinopathy patients [13]. Studies in Parkinson's disease (PD) patients with depression also reported SN‐related alterations [14, 15], affecting especially the insula [16, 17] and the cingulate cortex [18]. Interestingly, functional alterations involving the above‐mentioned networks have also been reported in major depressive disorder (MDD). The literature is rather consensual about the involvement of the DMN in depression [19, 20, 21]. Moreover, a meta‐analysis on large‐scale network dysfunction in MDD demonstrated FC disruptions involving the DMN, the FPN, the dorsal attentional network (DAN), the VAN and the affective network [19]. The SN [22, 23], and particularly the external connectivity of the anterior cingulate [24, 25] and the insula [26, 27, 28, 29], also seems to be involved in MDD symptomatology.

In continuity with our previous neuroimaging study [5], the present research aimed at investigating the FC changes associated with depressive symptoms in patients with prodromal or mild DLB. We believe that a better understanding of the functional brain features underlying depressive symptoms in DLB could both help in the detection of symptom‐specific biomarkers and lead to the development of new therapeutic strategies and targets. Based on the aforementioned literature data, we expected to find alterations mainly in within‐ and between‐network rsFC of the FPN, the DMN and the SN. Additionally, we assumed that we would observe rsFC changes in frontal, temporal and occipital areas, based on the results obtained in our previous structural imaging study on depression in DLB patients [5]. Although our primary focus was on these networks and regions, we opted for a whole‐brain approach. We believed it was important to adopt a global perspective, as this is, to our knowledge, the first time such a study has been conducted. Our second objective was to explore FC changes associated with antidepressant treatment in patients with DLB, through a comparative analysis of antidepressant‐treated versus untreated patients.

## 2 Materials and Methods

### 2.1 Participants

Participants represented a subsample of DLB patients and healthy control subjects (HCS) previously included in a structural MRI study [5]. Briefly, all participants were recruited from the tertiary memory clinic of Strasbourg University Hospital, France. DLB patients met the revised DLB consensus criteria [1, 2] and underwent a clinical examination, including measurement of the four core features: fluctuations [30], hallucinations [31], features of parkinsonism [32] and RBD [33]. In addition, patients had to be in the prodromal or mild stage of the disease (Mini‐Mental State Examination [MMSE] score ≥ 20 [34]). From the original DLB sample (*n* = 83), 15 patients were excluded because of missing or unusable images (i.e., incomplete field of view or poor quality of the acquisition), and 2 patients were excluded because of excessive motion during the fMRI session. A total of 66 DLB patients and 18 HCS were included in this research (Table 1). This study was part of the larger cohort study AlphaLewyMA (https://clinicaltrials.gov/ct2/show/NCT01876459). All participants gave written informed consent for the study according to the Declaration of Helsinki, and the study was approved by the local ethics committee of East France (IV).

**TABLE 1:** Demographic and clinical characteristics of dementia with Lewy bodies (DLB) patients and healthy control subjects (HCS).

Characteristics	Group		Statistic test*, p*
	DLB (*n* = 66)	HCS (*n* = 18)	
Age (years)^a^	70.24 (9.55)	67.86 (7.93)	*t* = 0.966, *p* = 0.337
Gender (M/F)	26/40	9/9	*χ* ^2^ = 0.655, *p* = 0.418
Handedness (R/L)	60/6	18/0	*χ* ^2^ = 1.762, *p* = 0.184
EL (years)^a^	11.79 (4.03)	13.67 (2.38)	*U* = 427.0, *p* = 0.067
Stage of disease (pro/dem)	47/19	—	
MMSE score^a^	26.30 (2.67)	29.06 (0.96)	**U = 201.0, *p* < 0.001**
Hallucinations (/9)^a^ ^,^ ^b^	2.05 (2.16)	0.11 (0.32)	**U = 998.0, *p* < 0.001**
Fluctuations^c^ ^,^ ^f^	6/18/17/19/6	12/5/1/0/0	**χ** ^**2**^ = **31.392, *p* < 0.001**
Akinesia^d^ ^,^ ^f^	28/35/3/0/0	17/1/0/0/0	**χ**2 = **15.4, *p* < 0.001**
Rigidity^d^ ^,^ ^f^	29/33/4/0/0	16/2/0/0/0	**χ** ^**2**^ = **11.558, *p* = 0.003**
Tremor^d^ ^,^ ^f^	60/6/0/0/0	16/2/0/0/0	*χ* ^2^ = 0.067, *p* = 0.796
RBD^e^ ^,^ ^g^	20/19/27	12/5/1	**χ** ^**2**^ = **10.217, *p* = 0.006**
MINI score^a^	2.29 (3.18)	0 (0)	
Psychotic phenomena (0/1) ^h^	47/19	18/0	
Antidepressant (0/1)	43/23	18/0	
Antipsychotics (0/1)	58/8	18/0	
Acetylcholinesterase inhibitors (0/1)	39/27	18/0	
Dopamine enhancers (0/1)	43/23	18/0	

*Note:* Significant *p* values (*p* < 0.05) are in boldface type.

Abbreviations: dem, mild dementia stage; DLB, dementia with Lewy bodies; EL, educational level; HCS, healthy control subjects; MINI, Mini International Neuropsychiatric Interview French version 5.0.0; MMSE, Mini‐Mental State Examination; pro, prodromal stage; RBD, rapid eye movement behaviour disorder.

^a^ Values are mean (SD).

^b^ According to [31].

^c^ According to [30].

^d^ According to the Unified Parkinson's Disease Rating Scale [32].

^e^ According to [33].

^f^ Rating from 0 to 4 (0/1/2/3/4).

^g^ Rating from 0 to 2 (0/1/2).

^h^ As measured by the fifth item of the hallucination scale [31].

### 2.2 Assessment of Depression

The depression screening score of the Mini International Neuropsychiatric Interview, French version 5.0.0 (MINI) [35] was used to assess the presence of depressive symptoms, as has already been done in recent studies on elderly subjects [36, 37, 38]. The MINI is a structured diagnostic interview exploring the main neuropsychiatric disorders, based on DSM‐5 criteria. The specificity and sensitivity of the scale have been acknowledged as sufficiently precise for diagnosing depression [39], and the scale appears to be well accepted by both patients and practitioners [40]. All questionnaire items are presented in Supporting Information S1. Patients are considered to have MDD if their score is ≥ 5/9; we considered a patient to have depressive symptoms if the score was ≥ 2. The questionnaire is designed so that only patients who answer “yes” to either of the first two questions (addressing depressed mood and anhedonia) are asked about the remaining seven items.

### 2.3 Neuroimaging Study

#### 2.3.1 MRI Data Acquisition

Images were obtained using a 3 T MR scanner (Verio 32‐channel Tim Siemens scanner; Siemens, Erlangen, Germany). A concomitant resting‐state blood oxygen level‐dependent (BOLD) and pulsed arterial spin‐labelling (ASL) sequence was used to acquire 121 whole‐brain T2*‐weighted (gradient echo) echo planar images (repetition time = 3 s; flip angle = 90°; echo time = ;21 ms; resolution = 38 × 64 × 28 voxels; field of view = 152 × 256 mm^2^; 4‐mm isotropic voxels). The first volume was intended for ASL assessment and was not considered for FC analyses. At the same session, a T1‐weighted three‐dimensional anatomical image was collected, using a volumetric magnetization‐prepared rapid acquisition with gradient‐echo (MPRAGE) sequence (repetition time = 1900 ms; flip angle = 9°; echo time = 2.52 ms; field of view = 256 × 256 mm^2^; resolution 256 × 256 × 256 voxels; slice thickness = 1 mm).

#### 2.3.2 MRI Data Preprocessing

Functional images were pre‐processed using the Statistical Parametric 12 package (SPM12, The Wellcome Trust Centre for Neuroimaging, London, UK) running on Matlab R2023a (MathWorks, Natick, MA, USA). The preprocessing steps applied included: filtering the ASL frequencies (low‐pass filtering at 0.112 Hz, based on [41]); slice‐timing correction; motion and B0 field inhomogeneity correction; co‐registration of the fMRI images to the T1‐weighted anatomical images; spatial normalisation to Montreal Neurological Institute space using the DARTEL approach, including smoothing with an 8‐mm full‐width at a half maximum Gaussian kernel.

### 2.4 Statistical Analyses

#### 2.4.1 Behavioural Analyses

Statistical behavioural analyses were performed using JASP software (https://jasp‐stats.org). To compare intergroup differences, we used Student *t* tests when the variables were normally distributed and non‐parametric Mann‐Whitney *U* tests when they were not. For categorical measures, *χ* ^2^ tests were applied. A threshold of *p* < 0.05 was used to determine statistical significance.

For fMRI analysis purposes, the DLB group was divided into two subgroups for comparison between patients with and those without depressive symptoms: depressed DLB (dDLB; MINI score ≥ 2) patients and non‐depressed DLB (ndDLB; MINI score < 2) patients. Similarly, DLB patients were divided into two other subgroups for comparison between patients with and those without antidepressant treatment: treated DLB (tDLB; use of antidepressants) patients and non‐treated DLB (ntDLB; non‐use of any kind of antidepressants) patients.

#### 2.4.2 Resting‐State Functional Connectivity Analysis

Functional connectivity analyses were performed using the CONN toolbox [42] running on Matlab R2023a. Thirty‐two regions of interest (ROIs) corresponding to the main nodes of the DMN, the FPN, the SN, the DAN, the language network (LN), the sensorimotor network (SMN), the visual network (VN), and the cerebellar network (CN) were selected from the “network atlas” implemented in the toolbox [42]. We also implemented the frontal, temporal and occipital areas as ROIs in the analyses, using the Harvard‐Oxford Atlas provided in the toolbox [43].

The first step was to identify individual ROI‐to‐ROI functional connectivity matrices by computing bivariate Pearson's correlation measures between the extracted mean BOLD signal time courses of each pair of ROIs. For each participant, their 6 motion parameters obtained during the preprocessing were added as covariates of no interest, and mean signals in cerebrospinal fluid and white matter were regressed out. The correlation coefficients were converted to normally distributed scores using Fisher's transformation to improve normality assumptions of the second‐level analyses.

Individual matrices were then entered into a second‐level general linear model, corrected for age and gender, to carry out group comparisons. Antidepressant treatment and laterality were also added as variables of no interest for comparison between dDLB and ndDLB patients. The results were reported when significant at a false discovery rate (FDR) corrected threshold of *p* < 0.05 at the seed level whenever possible, or at an uncorrected threshold of *p* < 0.05 at the seed level when no result survived multiple correction (FDR correction).

Multiple regression analyses were also performed to examine the effect of rsFC on depression scores in the DLB group only. While the MINI score was our variable of interest, we added age, gender and antidepressant treatment as variables of no interest. The results were reported when significant at a false discovery rate (FDR) corrected threshold of *p* < 0.05 at the seed level whenever possible, or at an uncorrected threshold of *p* < 0.05 at the seed level when no result survived multiple correction (FDR correction).

## 3 Results

### 3.1 Behavioural Results

Table 1 shows the demographic and clinical characteristics of DLB patients and HCS. The two groups were well‐matched in terms of age, gender, handedness and educational level. However, HCS had a significantly higher MMSE score compared to DLB patients. Regarding clinical symptoms, the presence of DLB core features (fluctuations, hallucinations, akinesia, rigidity and RBD) was significantly higher in DLB patients compared to HCS, except for tremor. Additionally, 25 of the 66 DLB patients had depressive symptoms, and 23 of the 66 were taking an antidepressant, while none of the HCS had depressive symptoms as evaluated by the MINI or were on antidepressant medication. Twenty‐one of the 25 dDLB (84%) patients had MDD (MINI score ≥ 5/9).

Table 2 presents the demographic and clinical characteristics of dDLB and ndDLB patients. This separation was carried out as part of the rsFC analyses in DLB patients. The two groups were matched in terms of age, gender, educational level, stage of the disease and MMSE score. However, the proportion of left‐handed patients was significantly higher in the ndDLB group.

**TABLE 2:** Demographic and clinical characteristics of depressed DLB (dDLB) patients and non‐depressed DLB (ndDLB) patients.

Characteristics	Group		Statistic test*, p*
	dDLB (*n* = 25)	ndDLB (*n* = 41)	
Age^a^	69.88 (9.49)	70.45 (9.71)	*t* = −0.237, *p* = 0.814
Gender (M/F)	10/15	16/25	*χ* ^2^ = 0.006, *p* = 0.937
Handedness (R/L)	25/0	35/6	**χ** ^**2**^ = **4.024, *p* = 0.045**
EL (years)^a^	11.6 (4.55)	11.91 (3.74)	*t* = −0.293, *p* = 0.770
Stage of disease (pro/dem)	18/7	29/12	*χ* ^2^ = 0.012, *p* = 0.912
MMSE score^a^	26.04 (2.73)	26.46 (2.66)	*t* = 0.622, *p* = 0.536
MINI score^a^	6.04 (1.93)	0 (0)	
Psychotic phenomena (0/1)^b^	16/9	31/10	*χ* ^2^ = 1.021, *p* = 0.312
Antidepressants (0/1)	15/10	28/13	*χ* ^2^ = 0.470, *p* = 0.493
Antipsychotics (0/1)	20/5	38/3	*χ* ^2^ = 2.345, *p* = 0.126
Anticholinesterase inhibitors (0/1)	12/13	27/14	*χ* ^2^ = 2.048, *p* = 0.152
Dopamine enhancers (0/1)	13/12	30/11	*χ* ^2^ = 3.066, *p* = 0.080

*Note:* Significant *p* values (*p* < 0.05) are in boldface type.

Abbreviations: dDLB, depressed dementia with Lewy bodies; dem, mild dementia stage; EL, educational level; MINI, Mini International Neuropsychiatric Interview French version 5.0.0.MMSE, Mini‐Mental State Examination; ndDLB, non‐depressed dementia with Lewy bodies; pro, prodromal stage.

^a^ Values are mean (SD).

^b^ As measured by the fifth item of the hallucination scale [31].

Table 3 shows the demographic and clinical characteristics of tDLB and ntDLB patients. This separation was carried out as part of the rsFC analyses in DLB patients. The two groups were matched in terms of gender, handedness, educational level, stage of the disease and MMSE score. However, the mean age of the ntDLB group was significantly higher than that of the tDLB group.

**TABLE 3:** Demographic and clinical characteristics of treated DLB (tDLB) patients and non‐treated DLB (ntDLB) patients.

Characteristics	Group		Statistic test*, p*
	tDLB (*n* = 23)	ntDLB (*n* = 43)	
Age^a^	65.93 (8.72)	72.54 (9.27)	**t = 2.819, *p* = 0.006**
Gender (M/F)	8/15	18/25	*χ* ^2^ = 0.314, *p* = 0.575
Handedness (R/L)	21/2	39/4	*χ* ^2^ = 0.007, *p* = 0.935
EL (years)^a^	11.44 (4.45)	11.98 (3.84)	*t* = 0.517, *p* = 0.607
Stage of disease (pro/dem)	15/8	32/11	*χ* ^2^ = 0.619, *p* = 0.431
MMSE score^a^	25.78 (2.84)	26.58 (2.57)	*t* = 1.16, *p* = 0.250
MINI score^a^	2.87 (3.48)	1.98 (2.99)	*t* = −1.09, *p* = 0.280
Psychotic phenomena (0/1)^b^	18/5	29/14	*χ* ^2^ = 0.856, *p* = 0.355
Antipsychotics (0/1)	19/4	39/4	*χ* ^2^ = 0.920, *p* = 0.337
Anticholinesterase inhibitors (0/1)	11/12	28/15	*χ* ^2^ = 1.853, *p* = 0.173
Dopamine enhancers (0/1)	14/9	29/14	*χ* ^2^ = 0.285, *p* = 0.593

*Note:* Significant *p* values (*p* < 0.05) are in boldface type.

Abbreviations: dem, mild dementia stage; EL, educational level; MINI, Mini International Neuropsychiatric Interview French version 5.0.0; MMSE, Mini‐Mental State Examination; ntDLB, non‐treated dementia with Lewy bodies; pro, prodromal stage; tDLB, treated dementia with Lewy bodies.

^a^ Values are mean (SD).

^b^ As measured by the fifth item of the hallucination scale [31].

### 3.2 Functional Connectivity Analysis

#### 3.2.1 ROI‐To‐ROI Analysis

No significant differences were found between dDLB and ndDLB patients at the set FDR‐corrected threshold.

**FIGURE 1:**
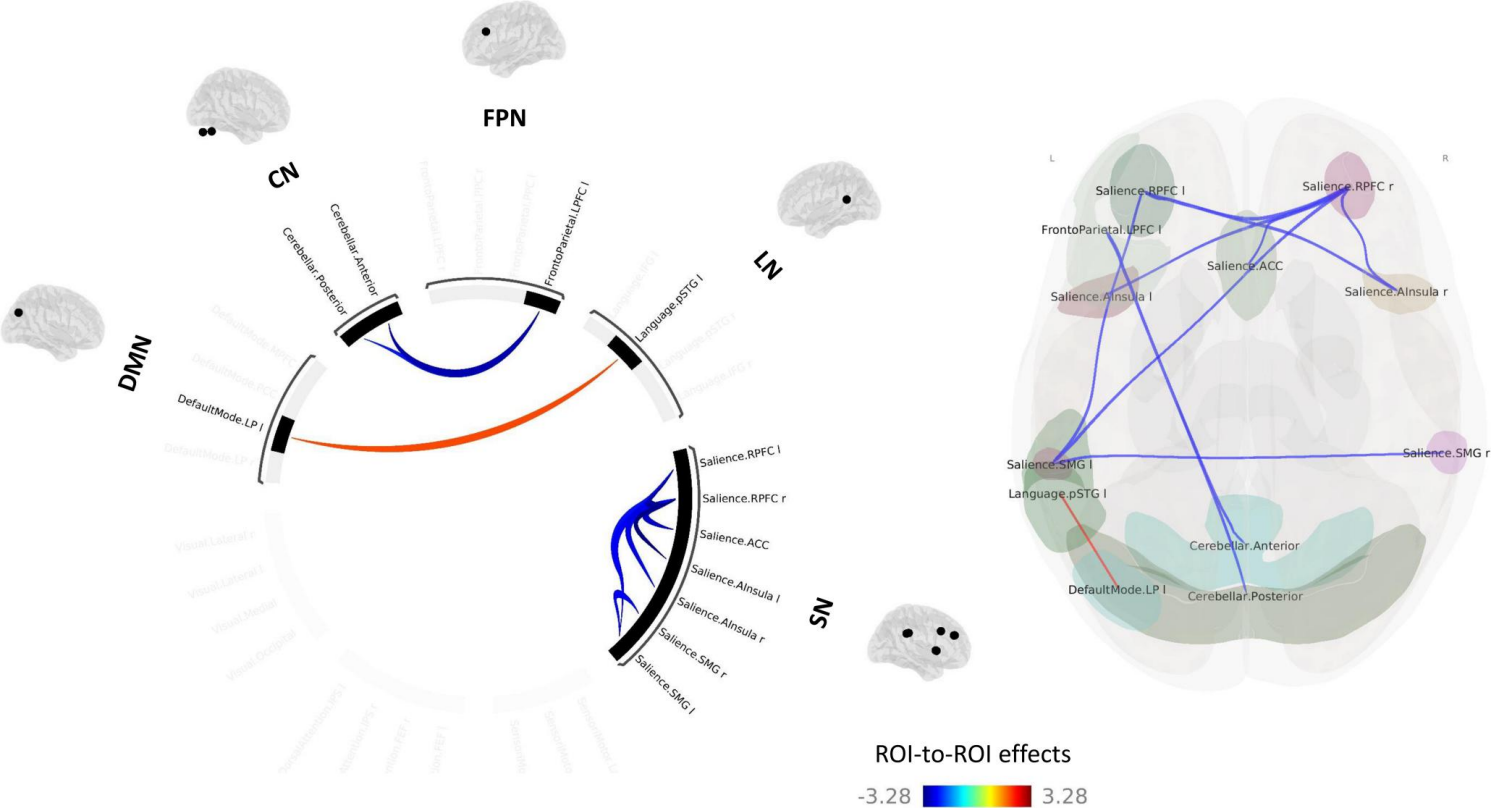
Between‐group differences in ROI‐to‐ROI functional connectivity: dDLB patients > ndDLB patients. ACC, anterior cingulate cortex; CN, cerebellar network; dDLB, depressed‐DLB; DMN, default‐mode network; FPN, fronto‐parietal network; LN, language network; LP, lateral parietal; LPFC, lateral prefrontal cortex; ndDLB, non‐depressed DLB; pSTG, posterior superior temporal gyrus; RPFC, rostral prefrontal cortex; SMG, supramarginal gyrus; SN, salience network. Results are represented at *p* < 0.05 (uncorrected) at the ROI level.

At an uncorrected threshold of *p* < 0.05, a significantly decreased FC was observed between a few ROIs within the SN in the dDLB group compared to the ndDLB group (Figure 1). The dDLB group also showed a decreased FC between the cerebellar network (anterior and posterior) and the lateral prefrontal cortex (LPFC) of the left FPN compared to the ndDLB group. In contrast, an increased FC was found between the left lateral parietal cortex (LPC) of the DMN and the left superior temporal gyrus (STG) (posterior part) of the LN in dDLB patients compared to ndDLB patients.

No significant differences were found between tDLB and ntDLB patients at the set FDR‐corrected threshold.

**FIGURE 2:**
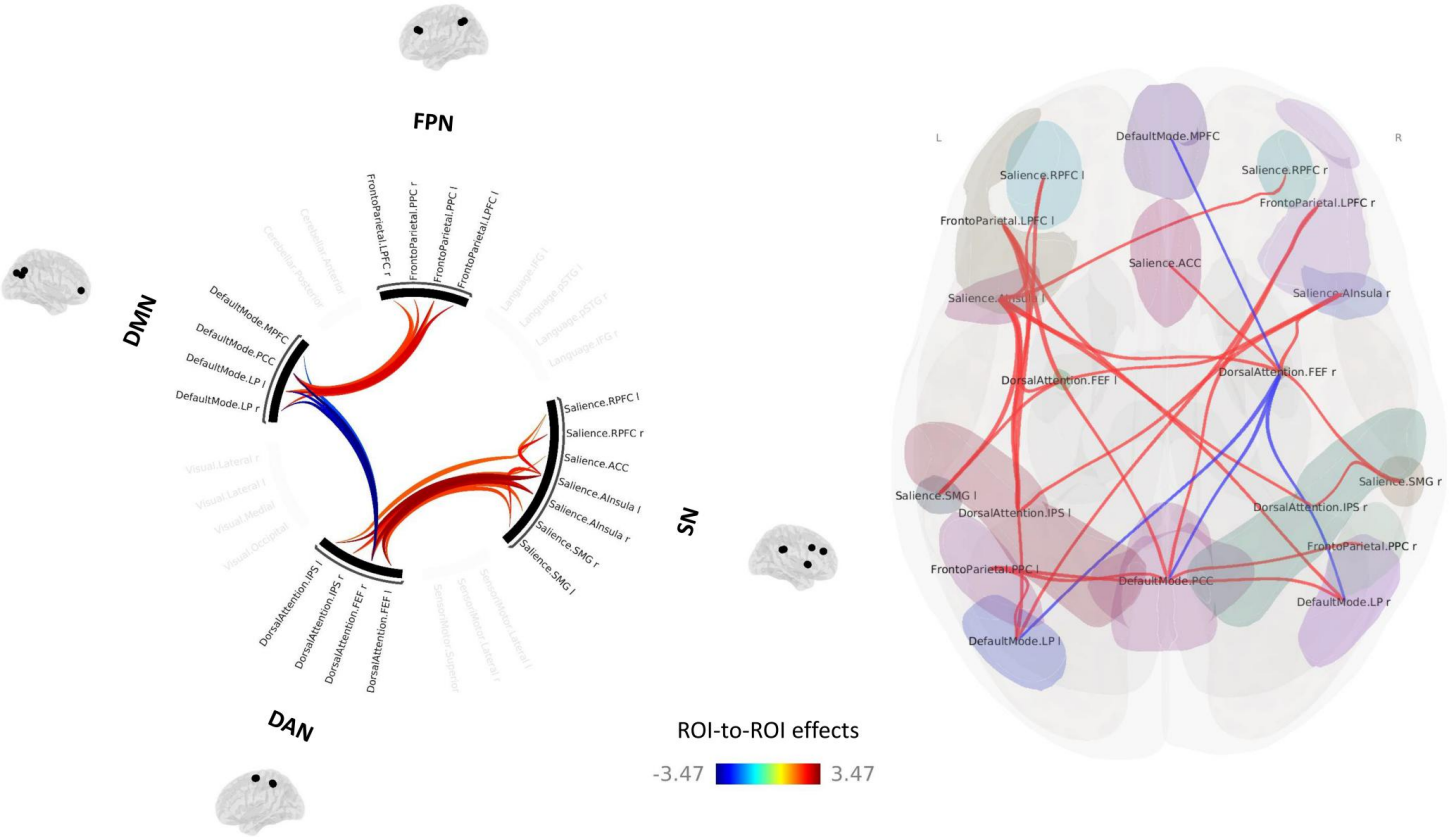
Between‐group differences in ROI‐to‐ROI functional connectivity: tDLB patients > ntDLB patients. ACC, anterior cingulate cortex; DAN, dorsal attentional network; DMN, default‐mode network; FEF, frontal eye field; FPN, fronto‐parietal network; IPS, intraparietal sulcus; LP, lateral parietal; LPFC, lateral prefrontal cortex; MPFC, medial prefrontal cortex; ntDLB, non‐treated DLB; PCC, posterior cingulate cortex; PPC, posterior parietal cortex; RPFC, rostral prefrontal cortex; SMG, supramarginal gyrus; SN, salience network; tDLB, treated DLB. Results are represented at *p* < 0.05 (uncorrected) at the ROI level.

At an uncorrected threshold of *p* < 0.05, the tDLB group had a significantly increased FC within a few ROIs of the SN, between ROIs of the SN and the DAN, and between ROIs of the FPN and the DMN compared to the ntDLB group (Figure 2). The tDLB group also showed a decreased FC between ROIs of the DMN and the right frontal eye field (FEF) of the DAN compared to the ntDLB group.

#### 3.2.2 Association Between FC Measures and Depression Scores in DLB Patients

**FIGURE 3:**
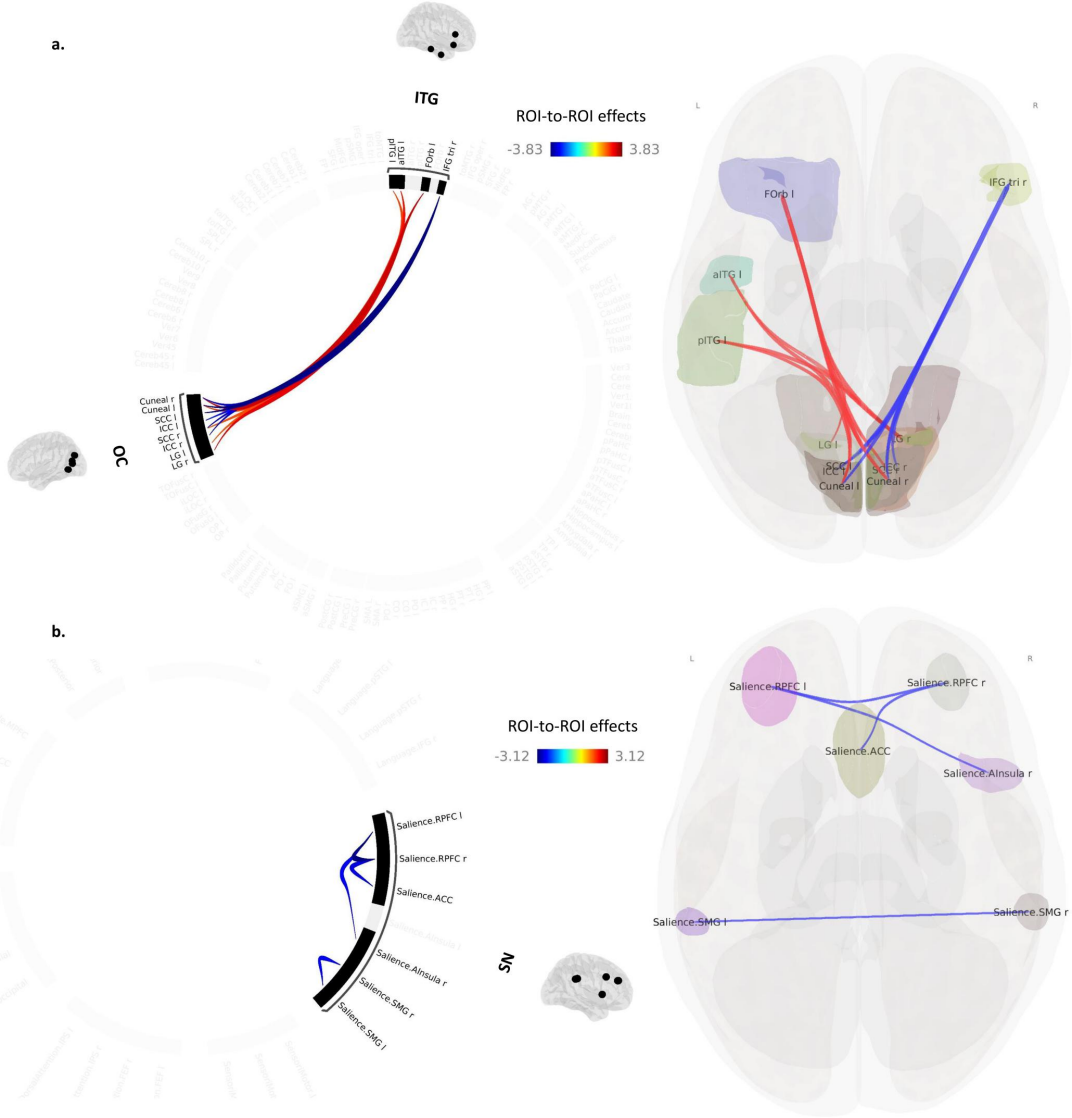
Effect of MINI scores on functional connectivity in the DLB group. (a) Results are represented at *p* < 0.05 (FDR‐corrected) at the ROI level. (b) Results are represented at *p* < 0.05 (uncorrected) at the ROI level. ACC, anterior cingulate cortex; DLB, dementia with Lewy bodies; Forb, frontal orbital; ICC, intracalcarine cortex; IFG, inferior frontal gyrus; ITG, inferior temporal gyrus; LG, lingual gyrus; OC, occipital cortex; RPFC, rostral prefrontal cortex; SCC, supracalcarine cortex; SMG, supramarginal gyrus; SN, salience network.

In the DLB group, a positive correlation was observed between patients' depression scores and the FC between the left orbitofrontal cortex (OFC) and the following occipital areas: the right and left cuneus, the right and left lingual gyrus, and the right supracalcarine cortex (*p* < 0.05, FDR corrected) (Figure 3a). Depression scores were also positively correlated to the FC between the left inferior temporal gyrus (ITG) (anterior and posterior) and occipital areas (i.e., between the left ITG [anterior part] and both the right cuneus and the right lingual gyrus, and between the left ITG [posterior part] and the left cuneus and the right lingual gyrus). Additionally, negative correlations were found between depression scores and FC between the right inferior frontal gyrus (IFG) (pars triangularis) and a few occipital regions (i.e., right and left cuneus, right and left intracalcarine cortex, right and left supracalcarine cortex).

At an uncorrected threshold of *p* < 0.05, depression scores were negatively correlated to the FC within the SN: between the right and left rostral prefrontal cortex (RPFC), between the right SN‐RPFC and the anterior cingulate cortex (ACC), between the left SN‐RPFC and the right anterior insula (AI), and between the right and left supramarginal gyrus (SMG) (Figure 3b).

## 4 Discussion

Our aim was to investigate FC changes associated with depression in patients with prodromal or mild DLB. Consistent with our hypotheses, we identified significant rsFC alterations in DLB patients with depressive symptoms.

This study highlighted the significant role of the rsFC between fronto‐temporal and primary visual areas in depression severity in DLB patients. Firstly, multiple regression analyses revealed that the severity of depressive symptoms in DLB patients was negatively correlated to the rsFC of the visual primary areas with the right IFG, suggesting dysfunctional top‐down processes in DLB patients with high depression scores. Indeed, the IFG is involved in cognitive control, and especially in inhibition and attentional regulation [44, 45, 46]. Depressed DLB patients could then experience difficulties in inhibiting or disengaging attention from negative visual inputs, as already suggested by Qiu and colleagues [47]. The authors especially mentioned that the disruption of visual primary cortex‐prefrontal connectivity they found in PD patients with depression could be linked to processing bias toward negative affective stimuli, in particular to selectively attend to negative information and experience difficulty to disengage attention from negative stimuli. Conversely, we hypothesised that higher MINI scores in DLB patients might be associated with a reduced ability to integrate visual information into higher‐order cognitive processes (i.e., planning, decision‐making processes, attention), which are often impaired in depression [48, 49, 50]. Secondly, patients' depression scores were also positively correlated to the rsFC between visual primary areas and both the left OFC and ITG. The OFC plays a fundamental role in emotional processing, decision‐making and affect regulation [51]. Moreover, the cuneus is structurally connected to the OFC [52], and would have a role in depression pathophysiology [53, 54]. Thus, we posited that an increased connectivity between the left OFC and the visual primary areas might reflect stronger processing bias and higher sensitivity towards negative visual stimuli. In other words, visual stimuli could be perceived more negatively, reinforcing the pessimistic interpretations and thoughts typically found in depression [55, 56, 57, 58]. Regarding the ITG, it has a significant role in semantic [59] and autobiographical memory [60, 61]. There is also evidence suggesting that the cuneus is involved in the retrieval of autobiographical memories [62]. Then, stronger connectivity between these two areas could indicate a tendency to ruminate, with visual stimuli triggering memories or thoughts tied to negative self‐perceptions or experiences.

Although not surviving multiple comparison correction, we also obtained interesting results that may reflect the involvement of the SN in depressive symptoms in DLB. The dDLB patients exhibited a reduced connectivity within the SN compared to the ndDLB patients. Moreover, the strength of rsFC within the SN was negatively correlated with the depression scores in DLB patients, suggesting that greater depressive symptomatology tend to be associated with weaker SN connectivity. This result aligns with the literature, which has recognised the role of SN‐related regions in depression [26, 27, 63, 64]. Similar decreases in SN connectivity have been associated with depressive symptoms in PD patients [14, 15, 17]. According to the triple‐network model [65, 66, 67, 68], the SN plays a critical role in switching between the internally focused DMN and the externally focused FPN, enabling the efficient allocation of attentional and cognitive resources depending on task demands. The complex interactions outlined in this model might be crucial to understand the emergence and persistence of depressive symptomatology [69]. Specifically, it has been suggested that difficulties in switching between the DMN and the FPN via the SN could compromise the brain's ability to disengage from self‐focused thoughts and adapt to external cues, leading to maladaptive self‐referential processing and emotional dysregulation [69, 70]. Consequently, we hypothesised that decreased rsFC within the SN in dDLB patients might contribute to difficulties in shifting attention from negative internal thoughts to external tasks, exacerbating feelings of rumination and lack of motivation. Furthermore, such results could help to explain why depression and DLB are intrinsically linked. As noted, the SN is frequently altered in both DLB and MDD patients. One could suppose that a personal history of depression could contribute to SN functional damage, operating as a potential risk factor for the development of DLB. Conversely, DLB is linked at an early stage to reduced SN rsFC, which may predispose individuals to the onset of depressive symptoms. However, further studies would be necessary to confirm such hypotheses. The use of dynamic rsFC analyses in DLB patients with depressive symptoms could particularly offer valuable insights into their brain's ability to switch between the DMN and the FPN over a given time course.

Our findings, though not strictly significant, also suggest potential disruptions in other FC networks in DLB patients with depressive symptoms. Notably, dDLB patients showed increased connectivity between the left LPC of the DMN and the left STG of the LN compared to ndDLB patients, consistently with a study showing a positive correlation between depression severity and FC between the DMN and the STG [71]. Given the DMN's role in ruminations [21] and the STG's involvement in language processing [72, 73, 74], this result may reflect the generation of self‐referential internal discourse and ruminations. Additionally, dDLB patients exhibited reduced connectivity between the CN and the LPFC of the left FPN compared to ndDLB patients. Although the cerebellum is not typically involved in mood or emotional disorders, several reviews and meta‐analyses have underlined its role in depression [24, 63, 75, 76, 77]. This last result could be interpreted in light of the ‘dysmetria of thought’ theory [78, 79], which posits that connectivity disruptions between the cerebellum and prefrontal and limbic cortices may lead to difficulties in regulating emotions [80, 81, 82, 83].

Regarding the impact of antidepressants on the rsFC in DLB patients, our comparison between tDLB and ntDLB patients tend to highlight rsFC changes in treated patients, as reported in most fMRI studies on antidepressants [84]. While our previous findings indicated that depression in DLB may be associated with decreased rsFC within the SN, we observed greater SN rsFC in antidepressant‐treated versus untreated patients. Antidepressant treatment might then contribute to a FC increase within this network and thus potentially improve the ability to shift between self‐referential thoughts and external stimuli, leading to better behavioural adaptation, improved emotional regulation and greater motivation. Although preliminary, our findings suggest that antidepressants may act on external FC of other networks such as the DAN, the DMN, the SN or the FPN. However, further research is needed to confirm these observations and clarify their clinical implications.

This study has several limitations. The main one in that most of our results are not corrected for multiple comparisons (i.e., FDR‐corrected). Although they are consistent with the literature, they will need to be replicated. Second, although our groups were matched for psychosis prevalence, it should be noted that psychosis may overlap with depression in the context of DLB, both in terms of clinical symptoms and FC hubs. Finally, the depression scale we used may lack sensitivity. Indeed, the MINI has been designed so that patients who answer “no” to the first two questions (looking at the two main depression criteria) are not asked the remaining items. We must also specify that the 2/9 threshold chosen to assess the presence of depressive symptoms has not been validated in the specific context of DLB or other neurodegenerative disorders.

## 5 Conclusion

These findings complement our previous structural study by providing critical insights into the functional brain features underpinning depressive symptoms in patients with DLB. Notably, we identified significant rsFC disturbances between fronto‐temporal and visual primary areas, which might be implicated in the manifestation of depressive symptomatology, especially contributing to difficulties in regulating emotions, processing biases towards negative stimuli, and self‐focused ruminations. Our findings should also encourage further research on the SN, as it might represent an important network for understanding the intrinsic link between DLB and depression.

## Ethics Statement

The study was approved by the local ethics committee of East France (IV).

## Consent

All participants gave written informed consent for the study according to the Declaration of Helsinki.

## Conflicts of Interest

The authors declare no conflicts of interest.

## Permission to Reproduce Material From Other Sources

This article does not include material reproduced from other sources.

## Supporting information

Supporting Information S1

## Data Availability

The data that support the findings of this study are available on request from the corresponding author. The data are not publicly available due to privacy or ethical restrictions.
