# Supplementary material for: Functional Connectivity Changes Associated With Depression in Dementia With Lewy Bodies
Source: Int J Geriatr Psychiatry. 2025 Feb 26;40(3):e70058. doi: 10.1002/gps.70058 (PMC11865007; doi:10.1002/gps.70058)
Supplement: Supplementary file 1 — Supporting Information S1 [file GPS-40-e70058-s001.docx]

**M.I.N.I.5.0.0 French Version**

| **1. Au cours des deux dernières semaines vous êtes-vous senti(e) triste, cafardeux(se), déprimé(e), la plupart du temps au cours de la journée et ce presque tous les jours ?** | OUI | NON |
| --- | --- | --- |
| **2. Au cours des deux dernières semaines, avez-vous presque tout le temps le sentiment de n'avoir plus goût à rien, d'avoir perdu l'intérêt ou le plaisir pour les choses qui vous plaisaient habituellement ?** | OUI | NON |
| 3. Votre appétit a-t-il notablement changé ou avez-vous perdu ou pris du poids sans en avoir l'intention (coter oui si +/- 5% du poids) ? | OUI | NON |
| 4. Avez-vous des problèmes de sommeil (endormissement, réveils nocturnes ou précoces, hypersomnie) presque toutes les nuits ? | OUI | NON |
| 5. Parlez-vous ou vous déplacez-vous plus lentement que d'habitude, ou au contraire vous sentez-vous plus agité(e) et avez-vous du mal à rester en place ? | OUI | NON |
| 6. Vous sentez-vous presque tout le temps fatigué(e), sans énergie ? | OUI | NON |
| 7. Vous sentez-vous sans valeur ou coupable ? | OUI | NON |
| 8. Avez-vous du mal à vous concentrer ou à prendre des décisions ? | OUI | NON |
| 9. Avez-vous eu à plusieurs reprises des idées noires comme penser qu'il vaudrait que vous soyez mort(e) ou avez-vous pensé à vous faire du mal ou au suicide ? | OUI | NON |

**M.I.N.I.5.0.0 : English Version**

| **1. For the past two weeks, were you depressed or down, or did you feel sad, empty or hopeless, most of the day, nearly every day?** | YES | NO |
| --- | --- | --- |
| **2. In the past two weeks, were you much less interested in most things or much less able to enjoy the things you used to enjoy, most of the time?** | YES | NO |
| 3. Was your appetite decreased or increased nearly every day? Did you weight decrease or increase without trying intentionally (i.e., by ± 5% of body weight)? | YES | NO |
| 4. Did you have trouble sleeping nearly every night (difficulty falling asleep, waking up in the middle of the night, early morning wakening or sleeping excessively)? | YES | NO |
| 5. Did you talk or move more slowly than normal or were you fidgety, restless or having trouble sitting still almost every day? Did anyone notice this? | YES | NO |
| 6. Did you feel tired or without energy almost every day? | YES | NO |
| 7. Did you feel worthless or guilty almost every day? | YES | NO |
| 8. Did you have difficulty concentrating, thinking or making decisions almost every day? | YES | NO |
| 9. Did you repeatedly think about death, or having any thoughts of killing yourself, or have any intent or plan to kill yourself? Did you attempt suicide? | YES | NO |
